# P31 Efficacy and safety of oral gepotidacin in the treatment of uncomplicated urinary tract infection: results of two randomized, multicentre Phase 3 trials (EAGLE-2 and EAGLE-3)

**DOI:** 10.1093/jacamr/dlad077.035

**Published:** 2023-08-02

**Authors:** Florian Wagenlehner, Caroline R Perry, Thomas M Hooton, Nicole E Scangarella-Oman, Helen Millns, Hwa-ping (Ed) Feng, Marcy Powell, Emily Jarvis, Jeremy Dennison, Amanda Sheets, Deborah Butler, John Breton, Salim Janmohamed

**Affiliations:** Justus-Liebig-University, Giessen, Germany; GSK, Collegeville, PA, USA; University of Miami, Miami, FL, USA; GSK, Collegeville, PA, USA; GSK, Stevenage, UK; GSK, Collegeville, PA, USA; GSK, Collegeville, PA, USA; GSK, Stevenage, UK; GSK, Brentford, UK; GSK, Collegeville, PA, USA; GSK, Collegeville, PA, USA; GSK, Collegeville, PA, USA; GSK, Brentford, UK

## Abstract

**Background:**

Uncomplicated urinary tract infections (uUTIs) are among the most prevalent infections worldwide. Currently, treatments are limited by antimicrobial resistance and antibiotic allergy/intolerance. There is an unmet need for new oral antibiotics active against uropathogens resistant to current treatments. Gepotidacin is a novel, bactericidal, first-in-class triazaacenaphthylene antibiotic that inhibits bacterial DNA replication by a distinct mechanism of action and binding site and provides well-balanced inhibition of two Type II topoisomerase enzymes. Gepotidacin is under investigation for treatment of uUTI.

**Methods:**

EAGLE-2 (NCT04020341) and EAGLE-3 (NCT04187144) were near-identical global Phase 3, randomized, parallel-group, double-blind, non-inferiority (10% margin) studies comparing the efficacy and safety of oral gepotidacin (1500 mg twice daily, 5 days) to nitrofurantoin (100 mg [Macrobid] twice daily, 5 days) for the treatment of uUTI. Eligible female patients were aged ≥12 years with ≥2 uUTI symptoms and urinary nitrite or pyuria (Figure 1). Per latest US FDA guidance, the primary endpoint, therapeutic response at test-of-cure (Days 10–13), was evaluated in patients with qualifying uropathogen(s) (≥10^5^ cfu/mL) susceptible to nitrofurantoin. Therapeutic success was combined clinical success (complete resolution of symptoms) and microbiological success (reduction of uropathogen[s] to &lt;10^3^ cfu/mL) without additional systemic antimicrobials. Data below are from a predefined interim analysis (IA) with efficacy/futility stopping criteria, conducted by an Independent Data Monitoring Committee (IDMC).

**Results:**

A total of 2986 randomized patients (EAGLE-2: *N*=1474; EAGLE-3: *N*=1512) were included in the IA. See Table 1 for key efficacy results available to IDMC that led to their recommendation to stop both studies for efficacy (non-inferiority) with no concerning safety findings.

**Table 1.** Therapeutic, clinical, and microbiological response at test-of-cure (data available to IDMC)

Endpoint	Gepotidacin 1500 mg BID	Nitrofurantoin 100 mg BID	Treatment difference (gepotidacin – nitrofurantoin) (95% CI)^a^
EAGLE-2	*N*=320	*N*=287	
Therapeutic success	162 (50.6%)	135 (47.0%)	4.3% (−3.6%, 12.1%)
Clinical success	210 (65.6%)	187 (65.2%)	1.2% (−6.3%, 8.7%)
Microbiological success	232 (72.5%)	194 (67.6%)	5.2% (−2.1%, 12.5%)
EAGLE-3	*N*=277	*N*=264	
Therapeutic success	162 (58.5%)	115 (43.6%)	14.6% (6.4%, 22.8%)
Clinical success	188 (67.9%)	167 (63.3%)	4.4% (−3.5%, 12.3%)
Microbiological success	200 (72.2%)	151 (57.2%)	15.0% (7.2%, 22.9%)

Study success rules were based on pre-defined interim analysis rules. Both studies were stopped for non-inferiority as the observed Z statistics of 3.5554 (EAGLE-2) and 5.8838 (EAGLE-3) were greater than the non-inferiority boundaries of the respective studies (2.065 for EAGLE-2 and 2.098 for EAGLE-3). Following pre-specified hierarchical testing strategy, superiority was then demonstrated in EAGLE-3 (observed one-sided *P* value of 0.0003 less than the superiority boundary [0.018]).

Calculated using Miettinen–Nurminen test adjusting for age and recurrence history.

These endpoints were evaluated in patients with all qualifying uropathogens determined to be susceptible to nitrofurantoin.

BID, twice daily.

**Conclusions:**

In two large Phase 3 trials using the latest, stringent, FDA efficacy endpoint, gepotidacin demonstrated non-inferiority in one trial and superiority in the other versus nitrofurantoin in the treatment of uUTI with an acceptable safety and tolerability profile. These data will be further described. Gepotidacin has the potential to be a useful oral antibiotic to treat uUTI and its evaluation in Phase 3 trials is an important step in addressing a significant unmet need.

**Figure 1.**
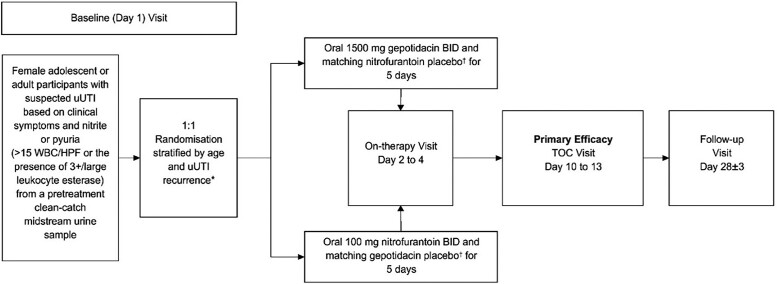
The efficacy of antibacterial gepotidacin evaluated trials (EAGLE-2 and EAGLE-3) study design. *Central randomization with stratification by age category (&lt;18 years, ≥18 to 50 years or >50 years) and uUTI recurrence (non-recurrent infection or recurrent infection, defined as a confirmed infection [not including the current infection in the calculation] with at least one prior episode within the past 3 months, at least two prior episodes within the past 6 months or at least three prior episodes within the past 12 months before study entry). ^†^Study treatment administered under double-blind, double-dummy condition. Each dose taken after food consumption and with water. BID, twice daily; HPF, high-power field; TOC, test-of-cure.

